# Comparison of Refractive Index Matching Techniques and PLIF40 Measurements in Annular Flow

**DOI:** 10.3390/s24072317

**Published:** 2024-04-05

**Authors:** Yago Rivera, Dorian Bascou, David Blanco, Lucas Álvarez-Piñeiro, César Berna, José-Luis Muñoz-Cobo, Alberto Escrivá

**Affiliations:** 1 IIE Instituto de Ingeniería Energética, Universitat Politécnica de Valencia, 46022 Valencia, Spain; yaridu@upv.es (Y.R.); dablade@upv.es (D.B.); lualpi@upv.es (L.Á.-P.); ceberes@iie.upv.es (C.B.); aescriva@iqn.upv.es (A.E.); 2 École Nationnale Supérieure d’Arts et Métiers, 75013 Paris, France; dorian.bascou@ensam.eu; 3 DEIOAC Departamento de Estadística, Investigación Operativa Aplicadas y Calidad, Universitat Politécnica de Valencia, 46022 Valencia, Spain

**Keywords:** conductance probes, annular flow, high-speed camera measurements, laser-induced fluorescence measurements

## Abstract

This paper investigates non-invasive techniques for annular two-phase flow analysis, focusing on liquid film characterization to understand the interfacial phenomena that are crucial for heat and mass transfer. Limited methods allow the study of the temporal and spatial evolution of liquid film, such as Planar Laser-Induced Fluorescence (PLIF). However, this method possesses optical challenges, leading to the need for improved techniques to mitigate refraction and reflection, such as Refractive Index Matching (RIM). This study utilizes an experimental annular flow facility to analyze both RIM and non-RIM PLIF over a range of liquid Reynolds numbers from 4200 to 10,400. Three configurations—PLIF RIM90, PLIF RIM40, and PLIF nRIM40—are compared from both qualitative and quantitative perspectives. In the quantitative analysis, key variables of the liquid film are measured, namely mean film thickness, disturbance wave height, and frequency. Variations in the analyzed variables indicate minor deviations, which are not likely to be caused by the technique used. However, all three methodologies exhibited errors that are estimated to be within a maximum of 10%, with a mean value of approximately 8%.

## 1. Introduction

Two-phase or gas–liquid flow is commonly encountered in industrial applications. In particular, annular flow is essential for effective heat transfer and fluid management in many processes, including oil transport, heat exchangers, boilers, condensers, nuclear reactors, chemical processing, geothermal energy, refrigeration, air conditioning, and cryogenic systems [1]. Increased knowledge of annular flow can help optimize pipeline transport, improve heat transfer in exchangers and steam systems, and affect chemical reactions in processing. Therefore, by understanding and controlling annular flow dynamics, industries can improve performance, energy efficiency, and safety in a wide range of applications. For example, annular flow is probably one of the most usual types of two-phase flows in fluid dynamics, particularly in scenarios like refrigeration systems, boiling water reactors (BWRs), and passive safety mechanisms such as passive containment cooling condensers in BWRs and certain types of small modular reactors (SMRs) [2,3]. Annular flow significantly affects heat transfer within these systems [4]. Fluid dynamics of two-phase flow systems refers to the research field that investigates and studies the behavior of two-phase fluids to improve their design and optimize facilities and installation. Annular flow has been a focus of study in two-phase flow for the past 50 years because the heat transfer in many two-phase systems takes place in this regime. A flow with a small amount of liquid moving through the walls of a conduct forming an annulus and with a gaseous core moving through it at a different velocity is defined as an annular flow. In general, depending on the relative velocity of the liquid film and the gas core, waves of different types form at the interface between the two phases [5]. In addition, the shear stress at the interface can tear off small drops from the crest of these waves, which are then dragged by the core gas flow [6]. In this regime, the liquid film contains small gas bubbles, while the core gas flow drags small droplets previously separated from the liquid film [4].

Accurately measuring the thickness of the liquid film using different sensors and techniques is crucial in the abovementioned applications. However, it can be challenging to get an accurate measurement due to the distortion caused by interfacial waves. There are various types of waves at the interface between the phases, with the most important being disturbance waves (DWs) and ripple waves (RWs), especially DWs [7,8,9]. The first type of waves, DWs, are characterized by having a large amplitude compared with the average thickness of the film, which is up to five times greater. They are periodic and constant throughout the section of the circular conduct. In contrast, RWs are more chaotic, and they are neither periodic nor exhibit coherent behavior through the pipe section.

**Figure 1:**
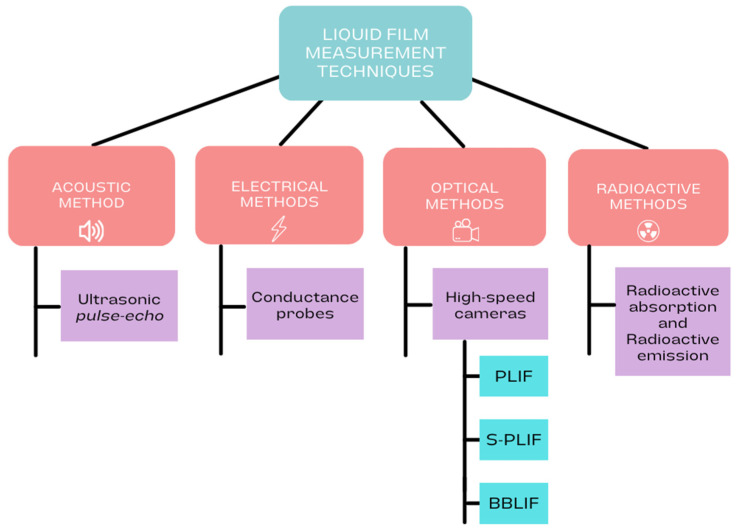
Summary of major techniques used to measure liquid film thickness experimentally.

Various methods have been explored and applied to address the difficulties in obtaining a quantitative measure of film thickness. An appreciable number of experimental methodologies have been employed over past decades for localized measurements at specific spatial positions while concurrently capturing temporal evolution. However, almost all of these methodologies can be classified into four major groups, although the most widely used are electrical and optical procedures [10,11]. Electrical techniques make it easy to obtain localized measurements with good spatial resolution. It is possible to vary the position of the measurement by relocating the probe to various positions within the installation [11,12,13,14]. One of the drawbacks of these intrusive methods is that, as the film thickness is disturbed by its interfacial waves, the probe device can disturb the flow at the measurement point; therefore, non-intrusive methods have been gaining ground in recent years. The main techniques used in film thickness measurement are summarized in Figure 1.

Conductance probes are the most commonly used electric method. In early work performed by Chu and Dukler [15,16], the authors measured the film thickness for flows of practical interest and different sizes of tubes. The interesting work of Ju et al. [17] is also worth mentioning. They also used this type of sensor in an 8 × 8 rod bundle and compared the measurements with flush-mounted conductance probes having two circular electrodes.

Non-intrusive methods include radioactive methods, such as those presented by Skjæraasen et al. [18], who employed X-rays to reconstruct the film thickness profile of an annular flow that could be applied for either two or three phases. A further example of a measurement method is that used by Wang et al. [19], who used an acoustic method. The ultrasonic echo resonance main frequency method is suitable for measuring thicknesses over particle image methods or conductance probes. However, electrical and optical procedures are the main methods used to obtain localized measurements at a given position and time.

Optical methods are modern techniques that are widely used to obtain results over time in a spatially distributed manner [20,21,22]. These non-invasive methods typically use high-speed cameras with high spatial resolution and can capture many frames per second. This allows the thickness of the liquid film in annular flow to be determined. These methods have evolved from localized techniques and have reached a high level of development. The use of optical methods is common due to their simplicity compared with other methods, and they provide a significant number of measurements. However, when applied to annular flow, they have some drawbacks, such as distortion caused by light reflection at the irregular interface between the two phases, gas and liquid, which is not uniform and contains many waves. In addition, the pipe wall can distort film images.

**Figure 2:**
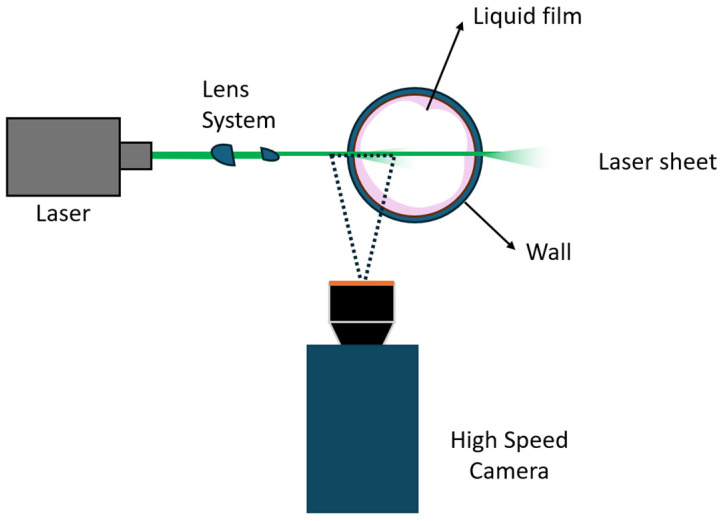
Schematic view of the PLIF technique for liquid film imaging, showing the basic position of the laser film created by the lens system and high-speed camera relative to the test section of the pipe.

One of the most utilized optical methods in contemporary research is Laser Induced Fluorescence (LIF). This method, along with its various techniques, is widely used. Employing this technique necessitates the introduction of a fluorescent substance into the liquid phase in the form of fluorescent dopant dye, typically Rhodamine B or 6G. Subsequently, this fluorescent substance is illuminated by a laser plane beam within the measurement region. Rhodamine B is an organic compound that fluoresces with an excitation wavelength $\lambda_{exc}=533\;nm$ and an emission wavelength $\lambda_{em}=627\;nm$. Similarly, Rhodamine 6G exhibits fluorescence with an excitation wavelength $\lambda_{exc}=530\;nm$, which is close to the second harmonic of the Nd—YAG laser at 532 nm, and an emission wavelength $\lambda_{em}=556\;nm$. The critical aspect of this technique lies in the wavelength difference between the incoming laser light and the emitted light from the fluorescent particles. By incorporating a filter at the digital high-speed camera to eliminate laser wavelengths, it becomes feasible to eliminate light rays originating from the laser source and entering the camera sensor. This process yields clear images of the liquid film [20]. Consequently, high-speed cameras can be utilized to capture images of the liquid layer, which is rendered distinguishable by the phenomenon of fluorescence explained above. The Planar Laser-Induced Fluorescence (PLIF) technique employs a laser sheet generated by a series of lenses, producing a planar laser sheet, as depicted in Figure 2.

The different researchers that used these techniques identified some limitations and difficulties, mainly in the transition from interpreting information from raw images to reaching the final variable tendencies and conclusions. To achieve this, researchers have used different methods based on LIF. Xue et al. [11,12,13] and Alekseenko et al. [22,23,24] have done exhaustive work in this area. In the applied method, the species excited by the laser beam will de-excite after only a few nanoseconds to microseconds and emit light at a wavelength larger than the excitation wavelength. Using a laser and its position relative to the high-speed camera determine the names to these sub-versions of LIF, such as PLIF.

Schubring et al. [25] used the PLIF methodology to provide direct visualization of the liquid film in upward vertical air–water annular flow and its image procession to reproduce the distribution of film heights. However, strongly curved interfaces relate to such flows where the two phases interact continuously. PLIF and Normal PLIF (PLIF90 or N-PLIF) present great challenges for liquid film thickness measurements in circular tubes. The two phases are in continuous interaction and present strongly curved interfaces, which act as mirrors. The refraction phenomenon results in several types of errors, as explained by Charogiannis et al. [26].

The PLIF method has been improved in recent years using two approaches. One of these is the Brightness-Based Laser-Induced Fluorescence (BBLIF) technique, developed by Alekseenko et al. [27]. This technique involves measuring the brightness of fluorescent light emitted by a dye in a liquid and converting it into film thickness. This is achieved through a relationship between brightness and thickness, considering factors such as absorption coefficient and interfacial reflection index and compensating for non-uniform laser illumination. BBLIF has been applied in cylindrical pipes and rectangular ducts with varying degrees of success. Although it is effective in measuring film thickness in flat regions, it is less sensitive in thicker regions and can be susceptible to errors from light reflections in complex or agitated flows.

A new technique called Structural Planar-Laser-Induced Fluorescence (S-PLIF) is currently being researched. This technique utilizes a structured light plane created using a Ronchi ruling plate. The Ronchi ruling plate is an optical device that has been developed with high precision, definition, and contrast. It is placed between the uniform laser light plane and the pipe, causing the light to arrive in parallel and alternating bright and dark streaks. By analyzing the gradient of these lines in the liquid film, it is possible to determine the true gas–liquid interface. Charogiannis et al. [26] developed a more complete version of this technique. In their paper, the authors address the main errors associated with the different techniques and how S-PLIF and the angle of inclination of the measurements help to reduce them.

Researchers have proposed new methods to improve the alignment between the laser and the camera, as it has a significant impact on measurement accuracy. One such method is the Acute PLIF (A-PLIF), as discussed by Tianyu et al. [28]. The authors use this nomenclature, although it is common to use PLIF plus the angle only (e.g., for 40°, it would be used PLIF40). The method involves placing both the incident laser sheet and the camera below the bottom interface of a plate, forming an acute laser optical path and camera alignment. Ting et al. [13] discussed the conventional PLIF90 method for annular flow measurements in axial water film and measurements where the angle between the laser and the camera was set to 40° (PLIF40). Other laser beam–camera alignments have also been proven effective, such as PLIF70 [11,12,13].

Rivera et al. [20] highlighted the challenges of refraction when measuring annular flow within a pipe. Their study mentions using the liquid box technique to mitigate these challenges. The above studies provide insights into different techniques used for measuring annular flow. Refractive Index Matching (RIM) has been widely used in optic measurements, as in Thorben et al. [29], who found that the method prevents optical distortion owing to light refraction and reflection at the interfaces by observing microscopic flow in a microchannel.

This paper presents a comparison between optical methods of measuring film thickness and interfacial wave characteristics at the CAPELON facility. This comparison helps to test the performance of each technique analyzed. Therefore, the advantages and disadvantages of each technique can be explored, as well as highlighting their potential limitations. The experimental mock-up is located at the Thermal-Hydraulics and Nuclear laboratory of the Institute for Energy Engineering at the Polytechnic University of Valencia. The experiments carried out consisted of the implementation and analysis of three variants of the PLIF method. In addition, the working fluid and the material of the test section have the same refractive index, which reduces the consequences of these undesirable optical effects. In the first technique, the test section is filmed directly using a high-speed camera. This method applies the commonly used 40° angle between the laser illumination and the camera (PLIF nRIM40). Recording with this angle is considered to minimize adverse optical phenomena, such as reflections and refractions at the air–water interface between or even the existence of the phenomenon of total reflection phenomena that hinder the subsequent image processing, as analyzed by Xue et al. [12,13,14]. In any case, due to the different values of the refractive index between the liquid film and the surrounding medium, the aforementioned phenomena of reflection and refraction of light mean that the measurements taken must be corrected [13,28]. The opposite is the case when a water box is placed between the test section and the camera so that the recording is carried out perpendicular to the illumination. Therefore, there are no optical phenomena that necessitate corrections of the measurements (PLIF RIM 90). However, for the precise positioning of the interface, reflections may occur at close positions owing to the liquid–air changes that the rays coming from the illumination may undergo in areas close to the measurement region; i.e., the so-called non-coherent interface error [26]. As will be discussed in detail, to minimize these phenomena, measurements can also be taken at an angle that differs from the right angle between the illumination and the camera, usually also 40° (PLIF RIM40). In this case, the correction to be made to the measurements will be motivated precisely by the existence of this viewing angle of 40° between the camera and the measuring section.

In accordance with the objectives described above, this paper is organized as follows: firstly, Section 2 describes the CAPELON facility, both the facility itself, including its instrumentation, and all aspects related to the PLIF measurements; Section 3 focuses on the description of the methodology used, particularly the PLF RIM90, PLIF RIM40, and PLIF nRIM40 configurations, along with the presentation of the experimental matrix, showing the image processing under all three configurations and providing an estimation of the major sources of experimental errors; Section 4 shows the results obtained using the three PLIF methods and provides a discussion of the results, analyzing their differences and trying to explain their causes; and finally, Section 5 displays the major conclusions, in which the advantages and drawbacks of each method are shown for the studied cases.

## 2. Experimental Apparatus

In this section, the specifications of the CAPELON facility are presented, wherein an experimental series has been conducted to compare various PLIF measurement techniques for the annular flow phenomenon. The equipment used to characterize mass flow rate and ambient and liquid temperatures is included. In addition, the instrumentation employed to perform measurements using different PLIF techniques will be detailed. Optical corrections of refraction and reflection effects, which are necessary to achieve accurate thickness measurements, will be addressed. This includes subpixel correction of the interfacial boundaries, as well as the angle between the laser and CMOS camera, aimed at minimizing errors induced by refraction and reflection, as documented in [13,30,31].

### 2.1. Instrumentation Used in the CAPELON Facility

The experimental facility CAPELON (CAracterización de PElícula ONdulatoria, Wavy Film Characterization in English) is specifically designed to generate downward annular flow under free-fall conditions. A liquid film descends attached to the walls of a tube, while a mass of air remains essentially stationary in the center. The phenomena reproduced resemble those found in steam generator tubes during certain accidental scenarios and are akin to the effects observed in passive cooling systems of third-generation reactors or SMRs. They are also manifested in various industrial processes involving heat transfer applications through shell-and-tube heat exchangers. In the experimental procedures of this study, there is no incorporation of heated fluids, which means that the heat transfer between the fluids or the wall is not considered. Results obtained using water as the working fluid at different mass flow rates will be presented.

**Figure 3:**
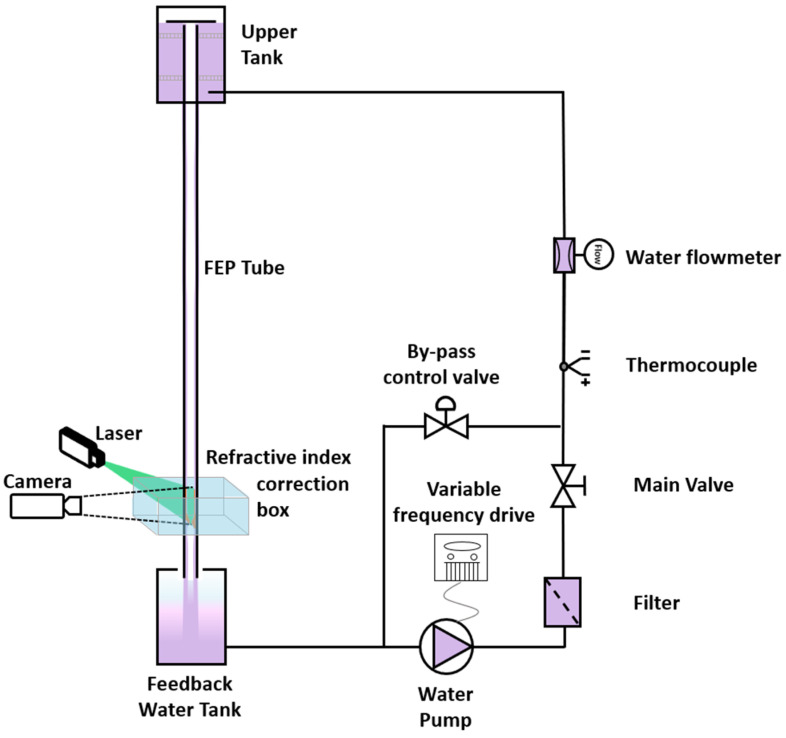
Schematic diagram of the CAPELON facility showing the PLIF measuring system.

The setup configuration of the CAPELON facility is depicted in Figure 3. In terms of dimensions, the length of the tube from the water injection point to the image capture section height is 2 m, with an inner diameter of 16 mm and an outer diameter of 19 mm. The tube material of the facility is fluorinated ethylene propylene (FEP), which shares the same refractive index as water. This material is selected to mitigate light refractions during camera captures. The circuit propels water using a pump controlled by a frequency inverter, which regulates the flow rate for conducting various experiments. Following the pump stage, corresponding sensors are installed to measure flow rate and temperature to ensure a controlled environment. In addition, several devices, such as regulating and safety valves, filters, and additional apparatus, are incorporated throughout the circuit to serve diverse functions. Water is directed toward the upper reservoir, which contains flow direction grids, permitting fluid movement solely in the vertical direction to prevent disturbances during overflow in the tube. The entrance section of the duct is precisely leveled horizontally. The lower reservoir is open, allowing air to escape and preventing water from backing up. From this point, the water is collected again by the pump to be recirculated.

To conduct measurements using the various PLIF techniques, a refractive index correction box is positioned at the measurement region at the same height as the high-spee camera and laser. This correction box is an open tank with a cubic geometric arrangement traversed by the tube. The purpose of this tank is to minimize refractions to prevent distortions in the photographs taken using the high-speed camera.

### 2.2. PLIF Setup

To conduct measurements using direct visualization systems, specifically employing PLIF techniques, several considerations and the characteristics of the experimental setup must be taken into account. Firstly, to mitigate refractions between the tube and the water comprising the annular flow, FEP is employed as the tube material. This is a specific material with almost the same refractive index as water. While water has a refraction index of 1.33, the FEP tube has a 1.34 refraction index (0.8% difference). Additionally, the water in the setup is dyed using rhodamine B, inducing a change in the wavelength of light emitted by the laser when traversing this medium. As the amount of rhodamine is negligible compared with the water, a change in the refraction index is not considered. The laser beam is directed with a very thin thickness by combining two lenses to capture the film section. A filter is applied in the camera to eliminate wavelengths below 580 nm, thereby removing the laser beam that has not passed through the water.

Among the three PLIF variants studied, the non-RIM technique requires an index of refraction adjustment. To avoid this problem, an index of refraction correction box filled with water is employed. This ensures that both the exterior and interior of the tube are filled with water, creating a homogeneous medium in terms of refractive index for light passage. Although there is air between the camera and the correction box, the perpendicular arrangement of both components prevents light deviation.

The high-speed camera used in this study features high-end technical specifications. The model is pco.dimax HD and has features that include a maximum frame rate of 2128 fps at full resolution and a minimum exposure time of 1.5 µs. It offers a maximum resolution of 1920 × 1080 pixels, and by reducing the Region of Interest (ROI), it achieves a maximum frame rate of 130,641 fps. The camera captures monochrome images with 16-bit tonal depth. In addition, the lens used has a focal length of 85 mm and a maximum aperture of f/1.4. The laser equipment utilized in this study operates at a wavelength of 532 nm and delivers a power output of 4 W. The specific model of the equipment is CNI MGL-N-532.

For the current experimental setup, the camera is configured with a frame rate of 2000 fps to capture sequences lasting 5 s. The ROI is set to an image size of 384 × 1080 pixels. The color temperature is adjusted to 5805 K, and the pixel depth range captured by the camera has been reduced to optimize the visible spectrum under operational conditions. This adjustment includes a 16-bit depth range where values ranging from 0 to 255 adequately capture information to determine contrast at the interface. To illuminate the liquid film, the laser equipment operates at 42% of its maximum power capacity.

## 3. Implementation and Analysis of PLIF Experimental Techniques

### 3.1. Configurations PLIF RIM90, PLIF RIM40, and PLIF nRIM40

**Figure 4:**
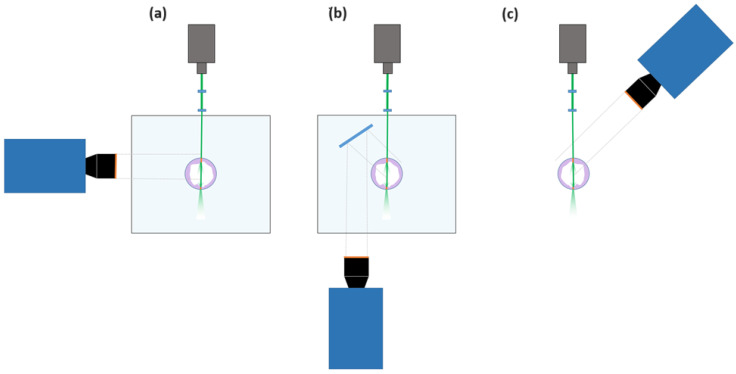
Setup of the three different configurations considered: (**a**) PLIF RIM90, (**b**) PLIF RIM40, and (**c**) PLIF nRIM40.

This section provides information about the different configurations explored, focusing specifically on RIM methodologies and their image processing. The primary objective is to discern the differences between RIM and non-RIM measurement approaches concerning the investigation of liquid film properties within curved geometries. Three different configurations with two distinct angle arrangements are considered in this study (Figure 4). These configurations comprise measurements conducted with a 90° angle setup between the laser source and the camera, denoted as PLIF RIM90, and another setup employing a 40° angle, designated as PLIF RIM40 and PLIF nRIM40. The selection of these angles is important, as it significantly influences the total reflection error of the measurements obtained [13]. The non-RIM technique is exclusively configured with a 40° angle. This configuration choice stems from the acknowledged complexities associated with refraction phenomena inherent in the measurement process. It is important to highlight that both measurement methodologies employing the 40° angle require a correction procedure to ensure accurate determination of the true film thickness. Moreover, non-RIM techniques impose an additional requirement for correction to account for the effects of refraction occurring within the liquid, tube, and air interfaces.

The experimental protocol involves capturing high-speed images of the liquid film within a defined ROI spanning 30 mm in height. The experimental conditions matrix considers different liquid Reynolds numbers, as described in Table 1. The frame-rate set for the camera is 2000 fps, and the total time of each run is 5 s. With these parameters, it is possible to obtain a high temporal resolution in which the propagating waves for the experimental matrix take a minimum of 40 images to travel along the ROI.

As previously mentioned, measuring with a different angle of 90° requires a proper correction between the measured or apparent film thickness and the true film thickness. When photons leave the liquid owing to fluorescence, they initially travel along the liquid film until they encounter the tube. To ensure undisturbed propagation, a FEP material with the same refractive index as water is employed. After crossing the tube, the photons encounter another interface, either with water for the RIM techniques or with air for the non-RIM technique.

In the case of RIM, angle correction can be directly obtained from the Pythagorean theorem, as no refraction occurs. In contrast, in the case of non-RIM, the adjustment requires the development of a series of equations based on Snell’s law and geometrical relations.

**Figure 5:**
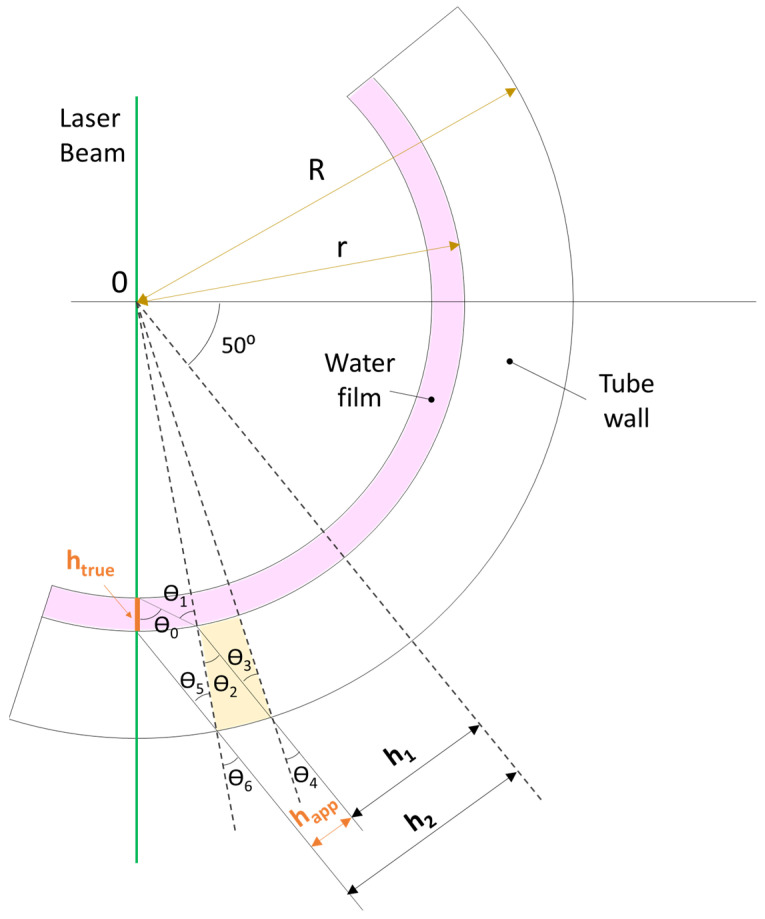
Schematic view of the photon’s path from the liquid film to the high-speed camera (based on Refs [13,20]).

Figure 5 provides a schematic view of the paths traversed by light coming from the interphase and from the liquid film close to the wall. As in this setup, a FEP tube with the same refraction index as water is employed, and the $\theta_{1}$ and $\theta_{2}$ angles are equal. For a generic case, whether there is RIM between the liquid film and wall, the main relationships derived are described in Equations (1)–(4) below, which consider the refractive index of the water, pipe, and air ($n_{w}$, $n_{p}$, and $n_{a}$ respectively). This equation system describes the relation between the thickness measured by the camera, or apparent film thickness, and the true film thickness. A deeper explanation of the procedure can be found in previous studies [13,20].

(1) $$R=\frac{r\sin\left(40{}^{\circ}-\theta_{6}+\theta_{5}\right)}{\sin\left(\theta_{5}\right)}$$

(2) $$\sin\left(\theta_{1}\right)=\frac{R}{r}\frac{n_{a}}{n_{w}}\left(\sin\left(\theta_{6}\right)-\frac{h_{app}}{R}\right)$$

(3) $$\begin{array}{cc} \sin\left(\theta_{0}\right)=sin & (40{}^{\circ}+{sin}^{-1}\left(\frac{R}{r}\frac{n_{a}}{n_{w}}\left(\sin\left(\theta_{6}\right)-\frac{h_{app}}{R}\right)\right)\\ & -{sin}^{-1}\left(\frac{R}{r}\frac{n_{a}}{n_{p}}\left(\sin\left(\theta_{6}\right)-\frac{h_{app}}{R}\right)\right)\\ & +{sin}^{-1}\left(\frac{n_{a}}{n_{p}}\left(\sin\left(\theta_{6}\right)-\frac{h_{app}}{R}\right)\right)-{sin}^{-1}\left(\sin\left(\theta_{6}\right)-\frac{h_{app}}{R}\right)) \end{array}$$

(4) $$h_{true}=r\left(1-\frac{\sin\left(\theta_{1}\right)}{\sin\left(\theta_{0}\right)}\right)$$

**Figure 6:**
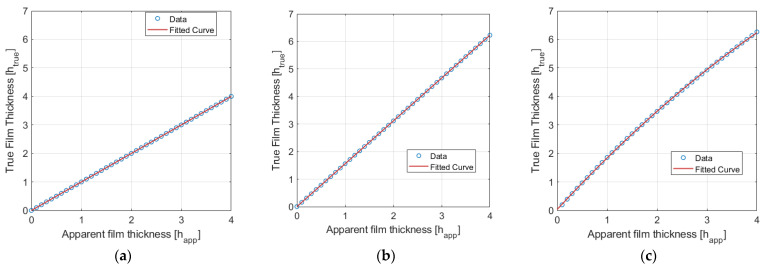
Relation between apparent and true liquid film thickness for the three different configurations: (**a**) PLIFRIM90, (**b**) PLIF RIM40, and (**c**) PLIF nRIM40.

Figure 6 illustrates the different relationships between apparent and true film thickness for PLIF RIM90, PLIF RIM40, and PLIF nRIM40. Figure 6a exhibits a direct relationship, as the camera is positioned perpendicular to the laser sheet. Figure 6b depicts a linear relation, as curvature effects are not considered, and only the angle between the camera and laser sheet perturbs the image. Finally, Figure 6c shows the outcome of combining the angle between the camera and laser with refraction through the media. In the last case, the relationship is not linear due to variations in the angles at which light from the liquid film impacts the side of the external tube, which depend on its thickness. Hence, curvature significantly influences the adjustment, which is dependent on the pipe’s radius.

Following this procedure requires iteration over the equations to obtain the true film thickness. Therefore, establishing a polynomial relationship proves optimal for expediting the process. Equations (5) and (6) represent the polynomial fits employed during image treatment for RIM40 and nRIM40 in millimeters.

(5) $${h_{real}}^{RIM40}=1.5566h_{app}$$

(6) $${h_{real}}^{nRIM40}=-0.0851{h_{app}}^{2}+1.8901h_{app}$$

### 3.2. Image Post-Processing

**Figure 7:**
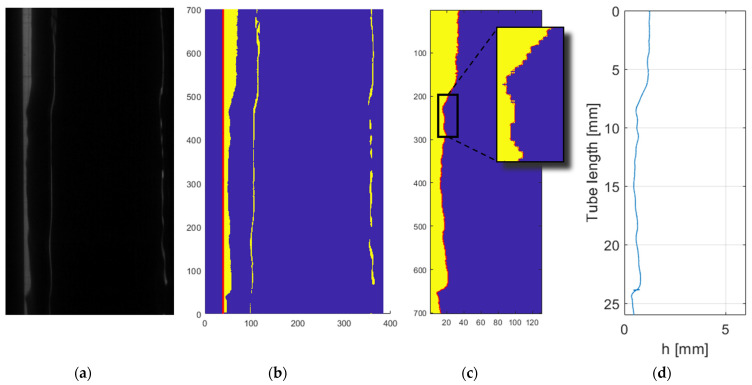
Processing steps of the snapshots: (**a**) Raw image; (**b**) wall detection and binarization; (**c**) image crop, sub-pixel detection showing the detail of the interface; and (**d**) final film thickness detected.

The high-quality images captured by the camera are post-processed to determine the interphase between liquid and gas as displayed at Figure 7. While seemingly straightforward, this process presents various challenges, thereby requiring adherence to a proper protocol. For this study, the following steps are executed:

1. Initially, Tagged Image File Format (TIFF) images are imported into MATLAB^®^ in grayscale.
2. The wall is detected during the calibration, but a first check is done to ensure that the camera has not been moved, considering that even small vibrations can cause a change of position of a few pixels. In addition, an algorithm evaluates its verticality.
3. Subsequently, a binarization process starts based on the image brightness.
4. The next step entails the removal of unnecessary portions of each snapshot to reduce the computational time required for image processing.
5. Employing the binarized image, the subpixel algorithm detects the location of the interface. This process employs Sobel filtering with 3 × 3 convolutional kernels. For a deeper understanding of this process, refer to [20].
6. Following interface detection, the film thickness is determined by applying the relationship between pixels and millimeters obtained from a calibration image at the onset of the runs.
7. Subsequently, the apparent film thickness undergoes correction to calculate the true film thickness (Equations (5) and (6)).
8. A moving mean filter is then applied, employing a window of 16 pixels to mitigate noise, particularly that arising from droplet detachment and deposition.
9. Lastly, the composition of the film over time is computed by processing all the snapshots.

### 3.3. Error Estimation

Two distinct error sources were evaluated to estimate the inherent errors in the measurements. The total error, denoted as $\varepsilon_{tot}$, includes both random and systematic errors. It can be computed as the squared sum of both sources, as expressed in Equation (7).

(7) $$\varepsilon_{tot}=\sqrt{{\varepsilon_{rnd}}^{2}+{\varepsilon_{sys}}^{2}}$$

To assess the systematic error, denoted as $\varepsilon_{sys}$, this study considers the impact of the conversion between pixels and real distance. This calibration process involves the installation of a target within the region of interest, which serves as a reference for extracting the pixel-to-millimeter conversion factor. Despite efforts to ensure optimal conditions, such as using a high-resolution target and achieving complete focus, it is challenging to assign a single precise value for pixels per millimeter due to inherent uncertainties. Following the calibration images taken at the beginning of each run, the potential deviation ranged between 25 and 27 pixels per millimeter. After processing the raw images to obtain the final film thickness, it was estimated that the average systematical error obtained was 7.2% for PLIF RIM90, 7.6% for PLIF RIM40, and 7.7% for PLIF nRIM40.

For the random error, denoted as $\varepsilon_{rnd}$, a series of 10 repetitive runs was conducted under various experimental conditions to compute the standard deviation. The accidental or random error is determined using Equation (8):

(8) $$\varepsilon_{rnd}=t_{n-1}^{\alpha /2}\frac{s_{n}}{\sqrt{n}}$$

Here, the equation employs the Student’s t quantile of α/2 for *n* − 1 degrees of freedom and the standard deviation of the variable values. Hence, using a t-table, the t value corresponds to 2.262 for a 95% confidence level and nine degrees of freedom. A concise summary table detailing the random, systematic, and total errors is provided in Table 2.

## 4. Results and Discussion

### 4.1. Results for the Temporal Evolution of the Liquid Film Thickness

This section presents the experimental data results obtained using each of the techniques described earlier; i.e., PLIF RIM90, PLIF RIM40, and PLIF nRIM40. RIM methods are commonly employed by the scientific community, although their implementation can be challenging for certain geometries. The use of FEP—or similar materials with a refractive index matching that of water, such as Perfluoroalkoxy Alkane (PFA) or Polytetrafluoroethylene (PTFE)—is often constrained by the limited sizes available in the commercial market and high prices. While alternative fluids with similar refractive indices to the geometry are feasible, they may yield results with liquid properties that differ from what is desired for specific studies. Therefore, a comparison between RIM and non-RIM methodologies can expose the drawbacks and opportunities associated with each technique and assist researchers in designing their own experiments.

**Figure 8:**
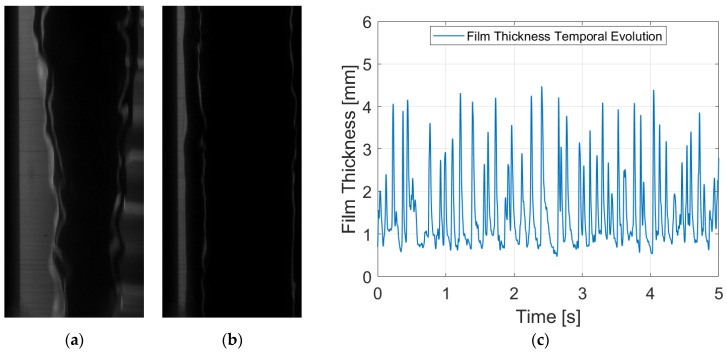
Different figures for PLIF RIM90 with a liquid Reynolds number of 7000: (**a**) Raw image with a disturbance wave passing through; (**b**) raw image of the film without the disturbance wave; and (**c**) temporal evolution of the liquid film thickness after treating the whole set run.

Among the configurations considered in this study, PLIF RIM90 yields snapshots with the highest spatial resolution. In this setup, the camera is positioned perpendicular to the laser sheet, enabling the capture of the illuminated liquid film with a greater number of pixels. Figure 8a,b depict two distinct snapshots showcasing the liquid film with and without a disturbance wave for a liquid Reynolds number of 7000. The raw images exhibit clarity and definition. Beyond the liquid–gas interface, a faint layer following the interface is observed, which likely results from the reflection of the laser sheet on the opposite side of the tube’s interface. However, this reflection is easily addressed during postprocessing, as no consistent information can be gathered from it. Figure 8c shows the temporal evolution of film thickness after processing 10,000 snapshots. Clear waves are discernible, and the signal is well-defined, facilitating the derivation of the figures of merit.

**Figure 9:**
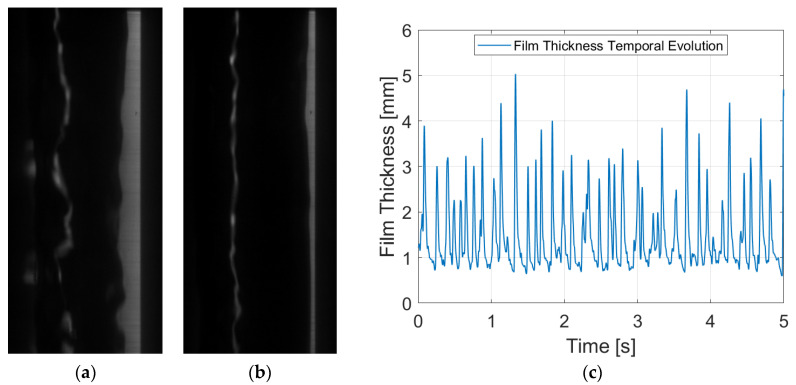
Different figures for PLIF RIM40 with a liquid Reynolds number of 7000: (**a**) Raw image with a disturbance wave passing through; (**b**) raw image of the film without the disturbance wave; and (**c**) temporal evolution of the liquid film thickness after treating the whole set run.

The PLIF RIM40 measurements (Figure 9) closely resemble those obtained with PLIF RIM90, as refraction is still not a factor. As explained in previous sections, employing a 40° angle allows the avoidance of non-coherent interface errors, which is particularly important when high fluid rates flow through the test section. This error is highly complex to observe and analyze [26], making PLIF RIM40 measurements compelling. This advantage itself compensates for the slight reduction in spatial resolution.

For qualitative comparison with other techniques, Figure 9a,b depict the raw images captured for snapshots with and without disturbance waves. Note that the liquid film is located on the right-hand side of the image. This happens because for PLIF RIM40, the light coming from the liquid film bounces through the mirror before going to the camera (Figure 4). The only discernible difference between the PLIF RIM40 and PLIF RIM90 images lies in the consistently smaller apparent film thickness. Upon correction, the images exhibit a similar behavior with a slightly diminished spatial resolution. Figure 9c illustrates the temporal evolution of the liquid film thickness, mirroring the behavior depicted in Figure 8c. This concurrence further remarks on the efficacy and reliability of PLIF RIM40 measurements in capturing temporal film dynamics.

**Figure 10:**
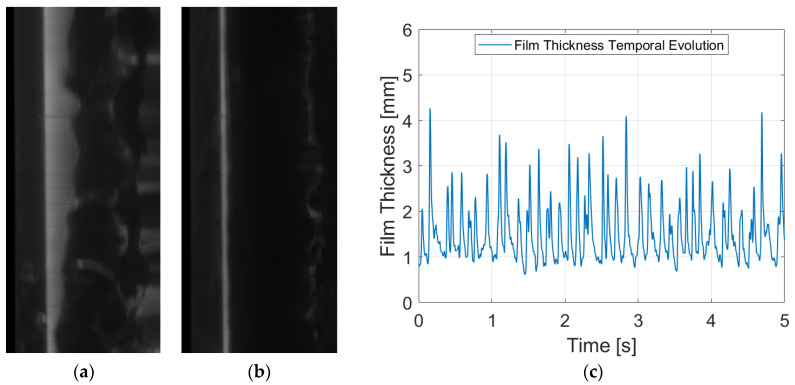
Different figures for PLIF nRIM40 with a liquid Reynolds number of 7000: (**a**) Raw image with a disturbance wave passing through; (**b**) raw image of the film without the disturbance wave; and (**c**) temporal evolution of the liquid film thickness after treating the whole set run.

Finally, PLIF nRIM40 measurements are one of the most interesting to compare in order to analyze the discrepancies with preceding RIM techniques. From a first impression of the snapshots (see Figure 10), it is noteworthy that the images exhibit a slight increase in distortion and blurriness, despite achieving full focus adjustment during the camera setup phase.

Figure 10a,b showcase raw snapshots for a liquid Reynolds number of 7000, with similar characteristics to those observed in previous techniques. Although images may appear marginally less defined, good resolution is achieved after post-processing the image. However, particular attention must be directed toward verifying the precise positioning of the wall after the initial calibration to identify any potential camera movement during the recording process. Figure 10c represents the liquid film for the entire run. Subtle disparities are discernible in the base liquid film, which is situated a few micrometers higher. Similar to PLIF RIM40, spatial resolution is diminished relative to RIM90 owing to the angle of observation. This observation is further corroborated in the error estimation calculation (see Section 3.3), where both techniques at 40° exhibit larger values of systematic error.

### 4.2. Results for the Figures of Merit of the Liquid Film

This paper is focused on annular flow, wherein the most important variables related to the hydrodynamic behavior are related to the liquid film thickness and the properties of the interfacial waves. To systematically evaluate the differences between the techniques employed, three primary figures of merit (FoM) take center stage: the mean film thickness $\left[h_{mean}\right]$, the disturbance wave height $\left[h_{DW}\right],$ and the disturbance wave frequency $\left[\nu_{DW}\right]$.

Mean film thickness is the simplest FoM; however, it is extremely important, as it provides the overall value of the film height for a specific liquid flow rate. This metric is derived by averaging the whole signal obtained for each individual run. In terms of disturbance waves, both their height and frequency are studied through the detection of peaks in the temporal evolution of the liquid film thickness. The average height of these peaks constitutes the disturbance wave height, while the transient statistic frequency is computed by quantifying the number of peaks throughout the entire duration of the run divided by the recorded time. It is important to note that frequency does not occur uniformly every second; instead, there are intervals with fewer waves scattered throughout and periods of heightened wave activity. In addition, the spacing between waves is inherently random and chaotic, as evidenced in Figure 8c, Figure 9c and Figure 10c. Notably, the widths of the disturbance waves are omitted in this study, as their relevance in comparing methodologies is minimal, given that refraction-induced distortions predominantly manifest in the radial direction.

**Figure 11:**
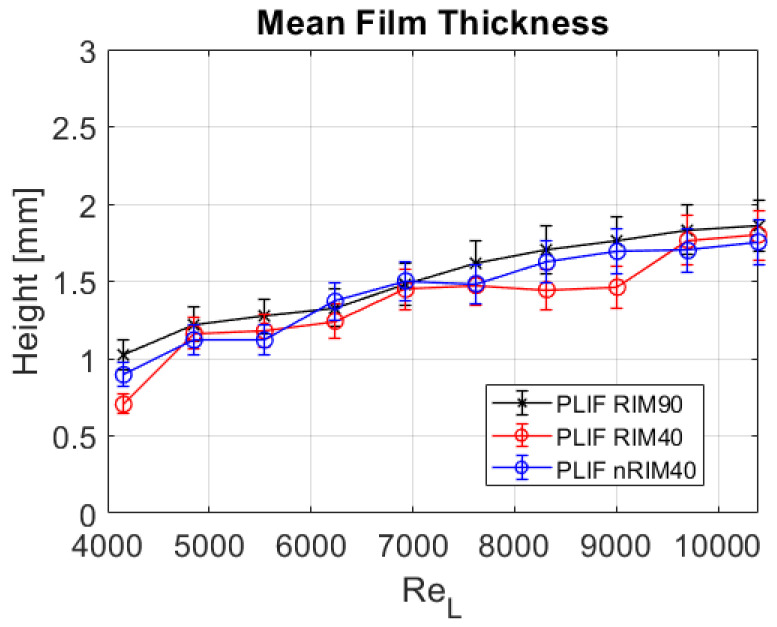
Mean film thickness $h_{mean}$ obtained for all measurements using the three techniques considered in this study.

Figure 11 illustrates the mean film thickness for various liquid Reynolds numbers, ranging from approximately 4000 to over 10,000. Without accounting for errors, upon initial inspection, all three techniques demonstrate consistency after appropriate postprocessing of the images. The range of average film thickness obtained spans from around 0.75 to 1.75 mm, with the obvious increasing tendency with the liquid mass flow rate. No discernible deviations attributable to the employed technique are evident.

The most significant discrepancies occur at liquid Reynolds numbers of 4200, 8300, and 9000 and are particularly notable in the PLIF RIM40 technique. In these instances, deviations reach up to 25% of the mean film thickness. However, these deviations do not consistently align with those obtained for consecutive liquid Reynolds numbers. For example, at a Reynolds number of 4900, the mean values obtained from different measurement techniques tend to converge. There is no observable systematic trend leading to a definite conclusion in terms of deviation between the techniques. Moreover, upon considering the errors inherent to optical methodologies outlined in Table 2 (such as the systematic error considered in this study), no clear drawbacks emerge when evaluating the mean film thickness.

**Figure 12:**
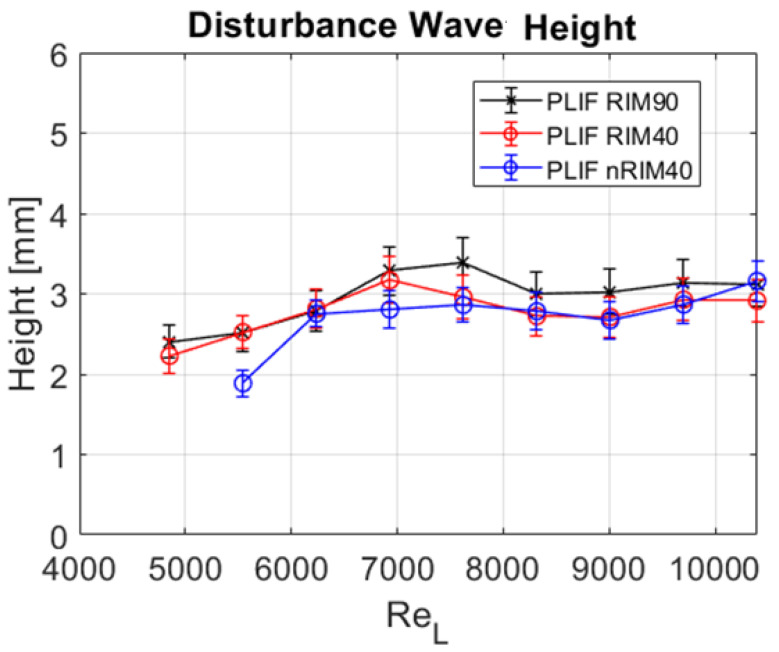
Disturbance wave height $h_{DW}$ obtained for all measurements using the three techniques considered in this study.

The disturbance wave height extracted from the entire dataset and across all three techniques is depicted in Figure 12. A slight increase in height is noted as the Reynolds number escalates. However, waves seem to plateau for the upper half of the liquid Reynolds rates, exhibiting minimal growth thereafter. Notably, for higher Reynolds numbers, namely 7500 and above, a reduction in wave height is observed in the two RIM techniques. However, this trend appears unrelated to the measurement methodology but rather to the inherent behavior of the waves after reaching a certain thickness where wall effects diminish. At the wall, the velocity is zero, and this reduction greatly influences the first layers of liquid. This is generally known as the boundary layer. As the thickness increases, the influence of the wall diminishes, allowing for different phenomena, such as the possibility of reaching higher velocities, which limits the thickness because even if the liquid flow increases, the thickness does not increase if the film’s velocity rises. Therefore, akin to the mean film thickness analysis, no significant discrepancies between the techniques are discernible from the disturbance wave analysis.

Furthermore, it is noteworthy that for Reynolds numbers lower than 4900, no disturbance waves manifest in the facility, indicating a laminar flow regime. In fact, the flow through the outlet of the test section for measurements at 4200 and below exhibited characteristics akin to a “frozen” laminar state. The transition from a regime without disturbance waves to one characterized by their presence occurs at a liquid Reynolds number of approximately 4500 to 5000. In this region, the appearance of waves naturally occurs as the flow rate increases. For the tests, we began with a flow rate of 0 before increasing the rate. In the case of PLIF nRIM40, the disturbance wave sub-regime was not initiated. Although repetition of the case could have been carried out until an eventual initiation occurred at the flow rate described, we decided to highlight the phenomenology found. This distinctive transition is evident in the measurements of PLIF nRIM40 depicted in Figure 12, where no disturbance waves are observed at ${Re}_{L}=4900$.

**Figure 13:**
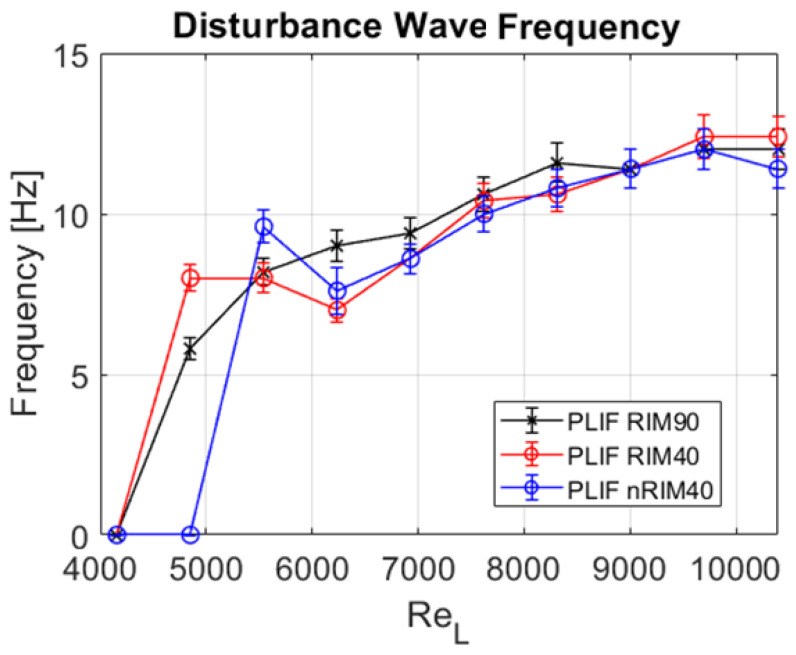
Disturbance wave frequency $\nu_{DW}$ obtained for all measurements using the three techniques considered in this study.

As mentioned earlier in this section, the disturbance wave frequency is a statistical variable calculated by dividing the total number of waves observed during the entire run by the recorded time. It offers a clear indication of the frequency of waves occurring throughout the run, although it should not be misunderstood as a constant frequency. Figure 13 shows the evolution of the disturbance wave’s frequency with the liquid flow. The observed differences in this variable are likely attributable to the sequential, non-simultaneous execution of the runs rather than discrepancies between techniques. The frequency starts at 0, indicating no disturbance waves, then increases rapidly to a value of around 8 before consistently rising with the liquid Reynolds number. As anticipated, no discernible differences stemming from the employed technique are evident.

To conclude, summing up all the evidence supports the conclusion that employing non-RIM techniques is both feasible and consistent when the proper procedures are followed. Attention to detail must be given to refractive index corrections, and ample knowledge of the liquid and materials used is required. While this study focuses primarily on the techniques themselves, a more in-depth examination of annular flow might require the utilization of supplementary methodologies for measuring liquid film thickness, such as S-PLIF, to mitigate potential obstructions (droplets, gas bubbles, light reflection objects, etc.) that could alter the interface between liquid and gas in snapshots. In addition, it is important to acknowledge that owing to the inherent challenges of optical techniques, errors for a setup exclusively dedicated to annular flow can still reach values of up to 10% when considering systematical errors (in the order of one- or two-pixel sizes) and random errors.

## 5. Conclusions

The focus of this paper lies in the comparative analysis of three optical techniques utilizing Planar Laser-Induced Fluorescence. The primary difference among the methodologies explored is the Refractive Index Matching, with one of the three techniques not complying with this requirement. Consequently, correction of the acquired images is necessary to ensure accurate measurement. An annular flow facility, CAPELON, is introduced to evaluate these techniques, complete with all required components to generate a downward liquid film for study. An overview of the three PLIF setups is provided, including two RIM configurations at 90° and 40° between the camera and laser, and the third employs a non-RIM approach at a 40-degree angle. A diverse range of liquid Reynolds numbers, spanning from 4200 to 10,400, is explored throughout the study. Based on Snell’s Law and geometric principles, the correction equation is described and applied to circular pipes.

Experimental data results obtained using PLIF RIM90, PLIF RIM40, and PLIF nRIM40 techniques are provided in the text, together with explanatory images. While RIM methods are widely employed in scientific research, their implementation can pose challenges, particularly in complex geometries. Despite slight disparities observed in specific cases, overall consistency is maintained across the techniques. The analysis of mean film thickness, disturbance wave height, and disturbance wave frequency show subtle differences, with variations being attributed primarily to flow conditions and hydrodynamic behavior rather than technique discrepancies. Error estimation based on both random and systematical errors shows inherent uncertainty in the measurements up to 10%. However, mean error values are close to 8.6% for the mean film thickness, 7.8% for the disturbance wave height, and 5.2% for the disturbance wave frequency. Considering all the analyses carried out, employing non-RIM techniques proves viable and reliable when the refraction distortion is properly corrected.

## Figures and Tables

**Table 1:** Experimental conditions used in this study.

Water Flow, $Q_{L}$lmin	Reynolds Number, ${Re}_{L}$	Superficial Velocity, $j_{L}$ms
3.0	4200	0.25
3.5	4900	0.29
4.0	5500	0.33
4.5	6200	0.37
5.0	7000	0.41
5.5	7600	0.46
6.0	8300	0.50
6.5	9000	0.54
7.0	9700	0.58
7.5	10,400	0.62

**Table 2:** Values for the error estimated in this study, showing the mean and maximum random, systematic, and total errors for each of the variables.

Figure of Merit	$\varepsilon_{rnd}[\%]$		$\varepsilon_{sys}[\%]$		$\varepsilon_{tot}[\%]$	
	Mean	Max	Mean	Max	Mean	Max
$h_{mean}$	1.5	1.6	7.5	7.8	7.6	8.0
$h_{DW}$	5.1	5.8	6.0	7.6	7.9	9.6
$\nu_{DW}$	6.0	7.3	0.0	0.0	6.0	7.3

## Data Availability

More data can be provided on request.

## Nomenclature

**Acronyms**	
A-PLIF	Acute Planar Laser-Induced Fluorescence
BBLIF	Brightness-Based Laser-Induced Fluorescence
BWR	Boiling Water Reactor
CAPELON	Facility Acronym of *Caracterización de Película Ondulatoria*
CMOS	Complementary metal–oxide–semiconductor
DW	Disturbance Waves
FEP	Fluorinated Propylene Ethylene
LIF	Laser-Induced Fluorescence
N-PLIF	Normal Planar Laser-Induced Fluorescence
nRIM	Non-Refractive Index Matching
PFA	Perfluoroalkoxy alkanes
PLIF	Planar Laser-Induced Fluorescence
PLIF40	Planar Laser-Induced Fluorescence at 40° angle
PTFE	Polytetrafluoroethylene
RIM	Refractive Index Matching
ROI	Region of Interest
FoM	Figure of Merit
RW	Ripple Waves
SMR	Small Modular Reactor
TIFF	Tagged Image File Format
**Variables**	
Re	Reynolds number
α	Significance level
h	Film thickness
$h_{app}$	Apparent film thickness measured by the high-speed camera
$h_{true}$	True film thickness
$h_{mean}$	Mean film thickness
$h_{DW}$	Disturbance wave height from the wall
$\nu_{DW}$	Disturbance wave frequency
$n_{w}$	Refractive index of water
$n_{p}$	Refractive index of the tube
$n_{a}$	Refractive index of the air
$j_{l}$	Superficial velocity of the liquid
θ	Angles of light refraction
